# The emerging role of the molecular marker p27 in the differential diagnosis of adrenocortical tumors

**DOI:** 10.1530/EC-13-0025

**Published:** 2013-08-29

**Authors:** Sofia S Pereira, Tiago Morais, Madalena M Costa, Mariana P Monteiro, Duarte Pignatelli

**Affiliations:** 1 Department of Anatomy and UMIB (Unit for Multidisciplinary Biomedical Research) of ICBASUniversity of PortoPortoPortugal; 2 Institute of Molecular Pathology and Immunology of the University of Porto (IPATIMUP)PortoPortugal; 3 Department of EndocrinologyHospital S.JoãoPortoPortugal

**Keywords:** adrenocortical tumors, StAR, IGF2, p27, Ki-67, adrenal cortex, adrenocortical carcinoma

## Abstract

Malignant adrenocortical tumors (ACTs) are rare and highly aggressive; conversely, benign tumors are common and frequently found incidentally (the so-called incidentalomas). Currently, the use of molecular markers in the diagnosis of ACTs is still controversial. The aim of this study was to analyze the molecular profile of different ACTs with the purpose of identifying markers useful for differentiating between these tumors. The ACTs that were studied (*n*=31) included nonfunctioning adenomas (ACAn)/incidentalomas (*n*=13), functioning adenomas with Cushing's syndrome (ACAc) (*n*=7), and carcinomas (*n*=11); normal adrenal glands (*n*=12) were used as controls. For each sample, the percentage area stained for the markers StAR, IGF2, IGF1R, p53, MDM2, p21, p27, cyclin D1, Ki-67, β-catenin, and E-cadherin was quantified using a morphometric computerized tool. IGF2, p27, cyclin D1, and Ki-67 were the markers for which the percentage of stained area was significantly higher in carcinoma samples than in adenoma samples. Ki-67 and p27 were the markers that exhibited the highest discriminative power for differential diagnosis between carcinomas and all type of adenomas, while IGF2 and StAR were only found to be useful for differentiating between carcinomas and ACAn and between carcinomas and ACAc respectively. The usefulness of Ki-67 has been recognized before in the differential diagnosis of malignant tumors. The additional use of p27 as an elective marker to distinguish benign ACTs from malignant ACTs should be considered.

## Introduction

Detection of adrenal tumors has increased in the last few years due to the widespread use of imaging methods such as computerized tomography or magnetic resonance imaging (1, 2). Most are benign nonsecretory adrenal tumors, with a prevalence of more than 4% in the adult population; in contrast, adrenocortical carcinomas (ACCs) are rare, having an incidence of between 0.5 and 2 per million, and are generally highly aggressive, with a poor prognosis that is expressed by a 5-year survival rate of between 16 and 38% (1, 3, 4, 5). The correct diagnosis of adrenocortical tumors (ACTs) is, therefore, understandably of growing importance.

ACTs can be divided into functioning and nonfunctioning tumors based on whether they secrete steroids or not (1). Functioning ACTs lead to various symptoms and syndromes depending on the secreted steroids, namely aldosterone, cortisol, or androgens with corresponding Conn's, Cushing's, or virilizing syndromes respectively (1). Some malignant tumors secrete precursor steroids or even inactive steroids and hence do not produce a clinical syndrome in spite of being functioning.

Differentiating between adrenocortical adenomas (ACAs) and ACCs is not always easy in spite of ACCs generally having rather larger tumor diameters and different histological characteristics. The most widely used methods are the Weiss scoring system based on nine histopathological characteristics and, more recently, the modified Weiss scoring system based on the five most reliable criteria (mitotic rate, abnormal mitosis, proportion of clear cells, necrosis, and capsular invasion), eliminating those considered to be more subjective or difficult to interpret (4, 6). Besides being difficult to assess, these parameters are still subjective and may not be enough to clearly define malignancy in every case of ACTs. In consequence, it is recognized by all specialists in this field that there is a need for additional tools for differential diagnosis of ACAs and ACCs (4, 7). This means that it is necessary to identify novel markers for classifying ambiguous ACTs as well as to understand their biological behavior.

Previous studies have already suggested that some molecular markers might be useful for defining malignancy in ACTs, namely markers involved in the cell cycle (4, 7, 8, 9, 10, 11, 12, 13, 14), cell adhesion (4, 7, 15, 16), steroidogenic regulation (7, 17, 18, 19, 20), and cell proliferation (4, 13, 14, 21, 22) and also growth factors (4, 7, 21, 22, 23). The reliability and accuracy of the studies using these markers have been questioned due to contradictory reports and, in many instances, subjectivity of quantification.

The main goal of this study was to search for putative molecular markers and analyze their presence and distribution in such a way that could improve the differential diagnosis of ACTs. We avoided the usual subjectivity in evaluation through the use of an automatic method and gathered a sample of significant size to test the efficiency of this method. In testing for biomarkers, we performed immunohistochemical labeling of 11 different molecules, namely those involved in steroidogenesis (StAR), regulation of the cell cycle (p53, p21, MDM2, p27, and cyclin D1), cell proliferation (Ki-67), and cell adhesion (E-cadherin and β-catenin), and the growth factor IGF2 and its receptor IGF1R.

## Subjects and methods

### Patients and tumors

Paraffin-embedded adrenal samples from a total of 43 patients were used. These included ACA samples (*n*=20) including samples of nonfunctioning adenomas (ACAn) (*n*=13) and adenomas with Cushing's syndrome (ACAc) (*n*=7) and ACC samples (*n*=11). In addition, 12 normal adrenal glands (NAGs) were used as controls. These were obtained from nephrectomy interventions as part of what used to be the normal surgical procedure for kidney tumors. A summary of the characteristics of patients is given in Table 1.

ACCs were diagnosed after surgical removal by histological analysis using the Weiss scoring system and only tumors with a score above four were included in the carcinoma group; ten patients developed disseminated disease and had a short survival time after diagnosis, compatible with malignancy. Only one patient is still alive, apparently disease free.

### Immunohistochemistry

Immunohistochemistry (IHC) was performed on formalin-fixed paraffin-embedded tissue sections mounted on adhesive microscope slides (HistoBond). The sections were successively deparaffinized, rehydrated in graded alcohols, and processed using the avidin–biotin immunoperoxidase method.

For antigen retrieval of IGF2 and MDM2, the sections were subjected to microwave treatment for 9 min and 15 min respectively in 0.01 M-citrate buffer at pH 6.0 with 0.05% Tween 20. For antigen retrieval of StAR and IGF1R, the sections were boiled for 3 min in 0.01 M-citrate buffer at pH 6.0 with 0.05% Tween 20.

Endogenous peroxidase was blocked with 3% hydrogen peroxide in methanol, followed by incubation with normal serum for 30 min. Then, the samples were incubated overnight at 4 °C with the appropriate primary antibodies: rabbit anti-human polyclonal antibodies to StAR (HPA023644; 1:100; Atlas Antibodies, Stockholm, Sweden), IGF2 (ab9574; 1:100; Abcam, Cambridge, UK), MDM2 (ab15471; 1:100; Abcam), and IGF1R (ab39675; 1:100; Abcam), The samples were then incubated with secondary antibodies at 1:200 dilution (polyclonal swine anti-rabbit, Dako, Glostrup, Denmark) for 30 min, followed by incubation with an avidin–biotin peroxidase complex (1:100; Vector Laboratories, Inc., Peterborough, UK) for 30 min. Diaminobenzidine was used as the chromogen and hematoxylin as the nuclear counterstain.

For the other markers, IHC was performed using the Kit Novolink Polymer Detection System (Leica, Wetzlar, Germany). For p53, p27, and E-cadherin, antigen retrieval was performed in a pressure cooker, after boiling, for 3 min, and for the markers cyclin D1, Ki-67, and β-catenin, it was carried out by treatment for 5 min in 0.01 M-citrate buffer at pH 6.0 with 0.05% Tween 20. For the marker p21, antigen retrieval was performed by incubation for 15 min in a microwave at 900 W. Endogenous peroxidase was blocked with 3% hydrogen peroxide in methanol. The sections were incubated overnight at 4 °C with the appropriate diluted primary antibodies: rabbit anti-human MABs to p53 (453M-94; 1:100; Cell Marque, Rocklin, CA, USA), p21 (421M-14; 1:50; Cell Marque), p27 (427M-94; 1:500; Cell Marque), cyclin D1 (271R-14; 1:500; Cell Marque), Ki-67 (27R-14; 1:100; Cell Marque), and E-cadherin (246R-14; 1:200; Cell Marque) and rabbit anti-human polyclonal antibodies to β-catenin (424A-14; 1:500; Cell Marque). Diaminobenzidine was used as the chromogen and hematoxylin as the nuclear counterstain. The following tissues were used for positive control: placenta for IGF2; colon carcinoma for p53 and Ki-67; breast cancer for IGF1R, MDM2, cyclin D1, and β-catenin; tonsil for p21 and p27, and lung adenocarcinoma for E-cadherin.

### Computerized image analysis

Using the camera AxioCam MRC Zeiss, ten photos were taken for each sample and antibody at 400× magnification, using the image acquisition software AxioVs40 v4.8.2.0 Zeiss for Windows, always under the same magnification and illumination and by the same researcher.

The images were analyzed using the image processing software ImageJ (originated at the National Institutes of Health, USA) with a color deconvolution plugin that can separate the stained area from the initial image and after which the software quantifies the percentage of the stained area. The percentage area stained corresponds to the percentage of the sample area that is specifically stained by the respective antibody. The ‘percentage area stained’ was compared between the different groups.

In the normal adrenal controls, only the staining of the zona fasciculata was included in the analysis, since the functioning adenomas included in this study were Cushing's syndrome cases deriving from these cells.

### Statistical analysis

Statistical analysis was carried out using GraphPad (LaJolla, CA, USA) Prism (version 5.00) for Windows, and a *P-*value <0.05 was considered significant. The comparison of two independent groups was carried out using Student's *t*-test. The one-way ANOVA test was used to compare three or more independent groups.

The diagnostic accuracy of the markers was evaluated using the receiver operating characteristic (ROC) curve. In a ROC curve, the true positive rate (sensitivity) is represented as a function of the false positive rate (1-specificity) for different cutoff points of a parameter. Sensitivity corresponds to the proportion of cases correctly identified by the marker as ‘carcinoma’, and specificity is the proportion of cases correctly identified by the markers as‘not carcinoma’.

ROC statistics allows one to make a correct decision regarding the best cutoff value for the differential diagnosis between benign and malignant cases by choosing the best sensitivity/specificity combination.

In summary, the area under the ROC curve (AUC) was used to measure how well a marker can distinguish ACAs and ACCs. Based on the AUC, the test was considered excellent between 0.90 and 1.00; good between 0.80 and 0.90; fair between 0.70 and 0.80; and poor between 0.60 and 0.70, and the test was considered to have failed if the value was below 0.60 (24).

The SPSS software (version 20.00) for Windows was used to evaluate the distribution of the β-catenin marker analyzed statistically through the *χ*^2^ square test and to evaluate correlations between the markers (through the Spearman test).

## Results

### StAR

**Figure 1:**
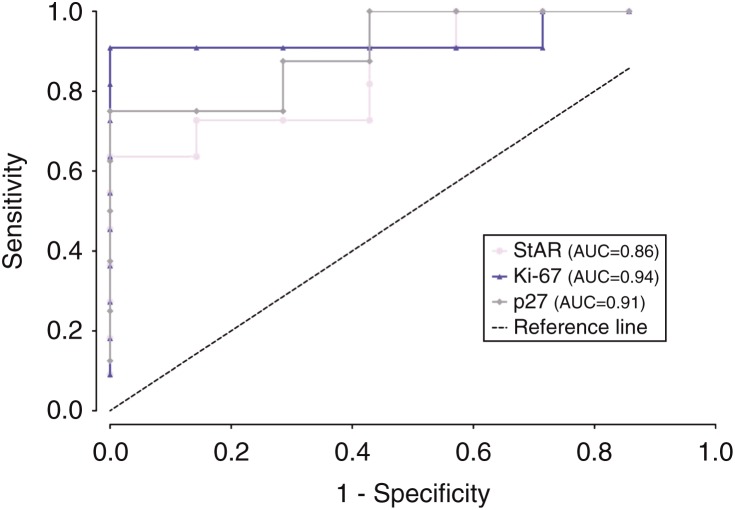
Graphical representation of ROC curves with the respective area under the curve (AUC) to compare carcinoma (ACC) and adenoma with Cushing's syndrome (ACAc) samples for the markers StAR, p27, and Ki-67.

StAR is a protein related to steroidogenesis often used to confirm that samples have an adrenal origin. In spite of some differences in staining, every sample was marked by the StAR antibody. Its staining was found to be higher in the NAG group than in all groups of altered adrenal tissues (Table 2). Comparison of data obtained from ACA and ACC samples revealed that there were significant differences only between ACAc and ACC samples (Table 2). Incidentaloma samples exhibited staining that was lower than that exhibited by ACAc samples and similar to that exhibited by ACC samples. The ROC curve was constructed to assess the accuracy of StAR for the differential diagnosis between ACAcs and ACCs. An area under the curve (AUC) value of 0.86 was obtained (Fig. 1).

### IGF2 and IGF1R

**Figure 2:**
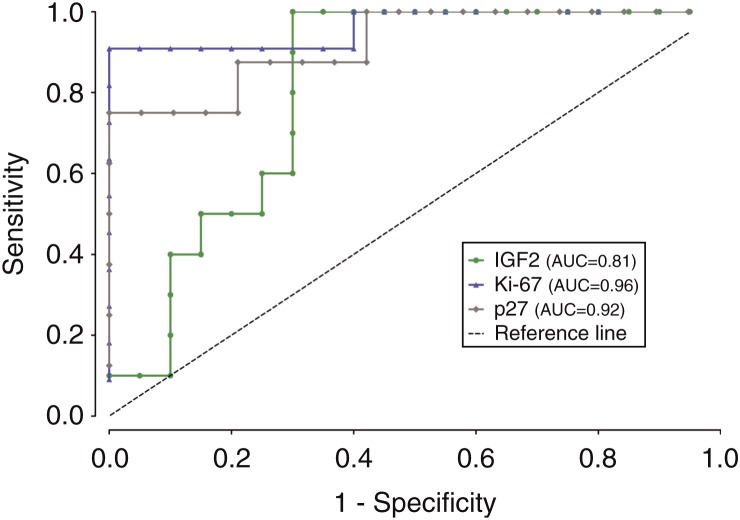
Graphical representation of ROC curves with the respective area under the curve (AUC) to compare carcinoma (ACC) and total adenoma (ACAt) samples for the markers IGF2, p27, and Ki-67.

**Figure 3:**
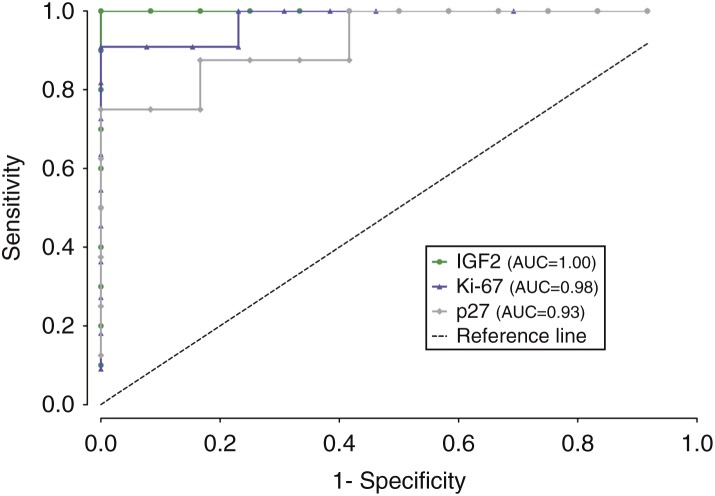
Graphical representation of ROC curves with the respective area under the curve (AUC) to compare carcinoma (ACC) and nonfunctioning adenoma (ACAn) samples for the markers IGF2, p27, and Ki-67.

IGF2 labeling of ACC samples was significantly higher than that of ACA samples (Table 2), with a ROC AUC value of 0.81 (Fig. 2). However, this difference was much more significant if one considered ACAn and ACC samples (Table 2), with an AUC value of 1.00 (Fig. 3) because ACAn samples exhibited almost no staining, while ACAc samples exhibited similar levels to ACC samples. The expression of IGF1R was not significantly different between the ACA and ACC groups.

### Cell cycle markers (p53, MDM2, p21, p27, and cyclin D1)

Of the five studied markers related to the cell cycle, a significant difference in labeling between ACA and ACC samples was only found for p27 and cyclin D1. ACC samples exhibited a significantly higher percentage area stained for p27, when compared with total ACA samples (Table 2), with an AUC value of 0.92 (Fig. 2). The same significant differences were found between ACC and ACAn samples (Table 2), with an AUC value of 0.93 (Fig. 3), and between ACC and ACAc samples (Table 2), with an AUC value of 0.91 (Fig. 1). For cyclin D1, significant differences were found between ACC and total ACA samples (Table 2), as ACC samples exhibited a significantly higher percentage area stained. On comparing ACAn and ACC samples, the difference was also found to be significant (Table 2); however, this marker exhibited an insufficient accuracy when the ROC curves were constructed with an AUC value of <0.80.

### Ki-67

The nuclear expression of the proliferation marker Ki-67 was significantly higher in ACC samples than in total ACA samples (Table 2), with an AUC value of 0.96 (Fig. 2). It was also higher than that in ACAn samples (Table 2), with an AUC value of 0.98 (Fig. 3), and than that in ACAc samples (Table 2), with an AUC value of 0.94 (Fig. 1). We also verified positive significant correlations between the percentage area stained for this marker with the percentages for the markers p27, cyclin D1, and IGF2 (*P*<0.01), with the highest correlation being observed between IGF2 and Ki-67 (correlation coefficient 0.64).

### Cell adhesion markers (E-cadherin and β-catenin)

No expression of E-cadherin was found in any of the samples. The staining for β-catenin exhibited different distributions, namely in the cell membrane, in the cytoplasm, and in the nucleus, and so this labeling could not be evaluated using the computerized system. In consequence, the distribution of this staining was evaluated by direct observation performed by two researchers independently, and results are presented in Table 3. The distribution of β-catenin immunostaining was significantly different between the groups (*P*<0.01). However, the abnormal location of staining, i.e., cytoplasm and/or nucleus, was not a marker for any specific group, as it was observed in ACC and ACA samples in spite of being present at different proportions.

## Discussion

Malignant ACTs are rare but highly aggressive and have a poor prognosis. Their prognosis is related to the tumor stage when the diagnosis is made, both clinically and by the pathologist (1). The differential diagnosis of benign (ACA) and malignant (ACC) tumors of the adrenal cortex is currently based on several histological parameters according to the Weiss scoring system, in which tumors with scores equal to or below two are classified as benign and those with scores equal to or above four as malignant. With regard to tumors with score 3, the Weiss scoring system might be insufficient to achieve a definite differential diagnosis between ACCs and ACAs (4). Everybody is in agreement that there is a need to identify novel molecular markers that will improve the differential diagnosis among ACTs and allow earlier identification of cases with malignant potential.

To meet this need, we performed an IHC investigation using 11 molecular markers, which were used to label a collection of samples from normal and pathological adrenal glands. These molecular markers have been previously studied separately by other research groups and were demonstrated to have potential usefulness for the differential diagnosis of ACTs. However, previous reports were sometimes either contradictory or subjective, mainly because results were analyzed using merely the researcher's observations (4, 13, 14, 21, 25).

One of the main strengths of our study was the use of a computerized evaluation method that allowed us to remove the subjectivity of the observer, and which could be used in future to study other molecular markers suggested by recent genomic studies (9, 26), which, if appropriately confirmed, may also become useful in clinical practice.

The employment of the ROC curves in our study was important, since it allowed us to determine the diagnostic accuracy of the molecular markers; compare the diagnostic accuracy of the different markers, and also calculate the best cutoff value to be used in the differential diagnosis of ACTs.

The main limitation of our study was the limited number of samples that we had access to; however, it could be a good starting point for large-scale studies, expanding the number of molecular markers but using this objective method of quantification.

Through StAR immunostaining, we confirmed that its expression was higher in functioning ACAs than in nonfunctioning ACAs, as expected. StAR is involved in a limiting step of steroidogenesis, the delivery of the precursor of steroid hormones, cholesterol, to the inner mitochondrial membrane, for the first enzymatic step in the steroidogenic pathway (20, 27, 28). Also as expected, the NAG group exhibited the highest expression of StAR. In contrast, ACC samples exhibited a lower expression of StAR compared with ACAc samples probably because the ACC group included more nonfunctioning tumors and also exhibited a generally decreased expression of StAR, possibly associated with its abnormal steroidogenesis. It was technically impossible to analyze the differences in the subgroups of functioning and nonfunctioning carcinomas due to the small number of cases in these subgroups, which resulted in a lack of statistical power. According to the ROC curve analysis, the accuracy of StAR as a marker for the differential diagnosis between ACCs and ACAcs has a high discriminative power, with an AUC value of 0.85. The cutoff value was calculated to be 8.26%.

We confirmed the growth factor IGF2 to be an excellent marker for differentiating between carcinomas and ACAn. This marker had an AUC value of 1.00, corresponding to 100% specificity and sensitivity for distinguishing ACAn from carcinomas using a cutoff value for the percentage of the stained area of 27.11. On the other hand, when comparing IGF2 data for total ACA vs ACC samples, we observed a lower AUC value, reflecting a lower accuracy for differential diagnosis. Soon *et al*. (22) reported a slightly higher AUC value than us (0.86 vs 0.81) by comparing ACA and ACC samples. A possible explanation for this difference is that in the study of Soon *et al*. (22) a lower percentage of ACAc were included (22), whereas we found IGF2 to be expressed in adenomas producing Cushing's syndrome. This finding will need confirmation with further research, as it has never been reported before. In conclusion, IGF2 has been proposed by many authors as a good marker for differentiating between ACAs and ACCs (21, 22, 29), but at least for the time being we suggest that its use be limited to the differential diagnosis between ACAns and ACCs.

No significant differences in the expression of the cell cycle molecular markers p53 (TP53), MDM2, and p21 (CDKN1A) were found between ACA and ACC samples. *p53* is a tumor suppressor gene and encodes a protein that promotes DNA repair (7, 10); MDM2 is a protein that inactivates p53 by binding to both the wild-type p53 and the mutated p53 protein (30, 31); and p21 is a cyclin-dependent kinase inhibitor (CDKi) induced by p53, which when overexpressed triggers cell cycle arrest in proliferating cells (10). Although no significant results were obtained for the p53 protein, we observed that some ACC samples exhibited a very high expression of this protein, which indicates the presence of *p53* mutations in these cases; however, other samples exhibited low expression, and it was this heterogeneity of p53 staining in the ACC samples that resulted in the difference between ACC and ACA samples for this marker not being significant.

The expression of cyclin D1, in contrast, was significantly higher in ACC samples than in ACA samples. Cyclin D1 is a regulator of the G1 to S phase transition of the cell cycle (32). Using the ROC curves to analyse the detection of differences between total ACA and ACC samples, we found an AUC value of <0.80, suggesting that this molecular marker is not very useful for the diagnosis of ACCs. Previous studies, using a cutoff of ‘5% positive cells’, failed to identify positive staining for this marker (13, 14).

The expression of Ki-67 protein was significantly higher in ACC samples than in ACA samples and NAGs. The ROC curve analysis for distinguishing between ACC and total ACA samples demonstrated an AUC value of 0.96 and the value of 0.50% as the best cutoff for the differential diagnosis of ACTs. Previous studies have reported similar results, and so the utility of Ki-67 is well supported (13, 14, 21, 22).

In this study, we could not associate the abnormal expression of β-catenin with the malignant character of the tumors, since we found nuclear expression in ACC samples as well as in nonfunctioning ACAs. Tissier *et al*. had already verified that abnormal expression was observed in both ACAs and ACCs and that most ACAs exhibiting abnormal β-catenin immunostaining were nonfunctioning ACTs, corroborating our results (4, 16).

E-cadherin, which is a protein of cell adhesion, generally reported to be associated with β-catenin, was not found in any of the studied samples, as has been described previously by Khorram-Manesh *et al*. (15).

In contrast, p27 immunostaining was the most novel positive finding of this study, since it allowed a clear distinction between ACCs and all other groups of tumors. The protein encoded by *p27* (CDKN1B) is a CDKi that regulates cell cycle progression from the G1 to the S phase of the cell cycle and upregulation of the expression of p27 results in cell cycle arrest and apoptosis (33). The percentage area stained for p27 was significantly higher for ACC samples than for all the other groups of samples. Analysis of the area under the ROC curve suggested that p27 has an excellent diagnostic accuracy for distinguishing between ACCs and both functioning and nonfunctioning ACAs with a value of 7.23% as the best cutoff for the differential diagnosis of ACTs. A previous study has shown the presence of p27 in almost all cases of ACCs, but failed to recognize its potential as a biomarker, since p27 was also observed in a substantial percentage of ACAs (13). Nakazumi *et al*. (25) had also already reported that the expression of p27 is increased in ACAs. However, both Nakazumi's and Stojadinovic's studies were carried out by direct observation by the researchers, rendering some level of subjectivity to the interpretation of immunostaining results, which we attempted to overcome by using an automated method of analysis. We also determined the percentage area stained, while the aforementioned studies measured the number of stained nuclei, which is a possible explanation for the differences in the results. An additional explanation for the discrepancy between the results may be the use of different primary antibodies in the studies (13, 25).

It must be pointed out that these two previous studies had reached contradictory conclusions, as in Nakazumi's study the difference in the percentage of stained nuclei between the ACA and ACC samples favored the marking of the benign tumors, and although statistically significant, this difference was less pronounced than the one observed in the more recent study carried out by Stojadinovic, which found increased p27 staining in malignant tumors, similar to that observed in our study (13, 25). By being far less subjective and defining more correctly the cutoff value through the ROC analysis, our method made the identification of the distinction between ACCs and ACAs very significant. Results that were similar to the results of our study have been described previously for breast tumors and melanomas (34, 35, 36).

The presence of high levels of a CDKi in ACC samples is somewhat counterintuitive. However, the positive results of several studies indicate the existence of a mechanism that allows some cancer cells to either have a tolerance phenomenon for this inhibitor of cell cycle progression or to develop the ability to repress the activity of p27 as an important step in tumor progression. This would mean that p27 could be present but would be unable to produce its usual actions to arrest the cell cycle. An alternative hypothesis is that *p27* gene could have mutations, resulting in a modified p27 protein that could have a still unknown role in tumorigenesis or tumor progression. However, *p27* mutations have been described as a very rare phenomenon in human cancer. Nickeleit *et al*. (36) suggested an interesting intuitive hypothesis, which states that if a tumor cell does not need to mutate a tumor suppressor gene, this might mean that the resulting protein must have some sort of a tumor-promoting function, even if so far unidentified.

Through correlation studies, we could verify that there were positive correlations between the levels of the growth factor IGF2, the cell cycle regulators p27 and cyclin D1, and Ki-67, meaning that the markers IGF2, p27, and cyclin D1 may all be promoting the high proliferative drive of ACCs. In our samples, only one case of ACC was negative for p27 and positive for Ki-67, while none of the cases positive for p27 were negative for Ki-67. Combining the AUC of Ki-67 and p27 did not produce any additional improvement in the ROC curve analysis, since each of these markers separately had already attained an excellent level of discrimination.

In conclusion, of the studied molecular markers, p27 and Ki-67 were the ones that demonstrated the highest discriminative power in differentiating between ACCs and ACAs, while IGF2 only seemed to be useful in differentiating between ACCs and ACAns and StAR for the differential diagnosis between ACCs and ACAcs. The main novel demonstrations of this study were the use of an automatic method of analysis to remove subjectivity and that p27 is overexpressed in ACCs, suggesting that this CDKi should have a still unknown role in adrenocortical tumorigenesis and possibly also represent a potential treatment target for malignant ACTs.

## Figures and Tables

**Table 1:** Demographics of the patients and clinical features of the tumors.

	**ACC** (*n*=11)	**ACA** (*n*=20)	**NAG** (*n*=12)
Median age, years (range)	46 (27–59)	49 (23–76)	49 (22–57)
Sex F:M	6:5	14:6	10:2
Presentation			
Cushing's syndrome	6	7	NA
Nonsecretory	5	13	NA
Weiss score	>4	≤2	NA
Tumor size (mm) (range)	188±98 (75–310)	38±23 (15–60)	NA

ACC, adrenocortical carcinoma; ACA, adrenocortical adenoma; NAG, normal adrenal gland; NA, field is not applicable.

**Table 2:** Percentage area stained for the different immunohistochemical markers (mean±s.e.m.) in the different groups.

	**ACC** (*n*=11)	**ACAt** (*n*=20)	**ACAc** (*n*=7)	**ACAn** (*n*=13)	**NAG** (*n*=12)	***P***
Cytoplasmic markers						
StAR	7.11±1.95^a^	10.48±2.03^b^	18.12±3.48^c,d,e^	6.02±1.40^f^	25.21±3.21	<0.01^a,d^
						<0.001^b,f^
						<0.05^c,e^
IGF2	35.31±1.33^a^	23.90±2.44^g^	35.73±1.75^d,e^	17.67±2.17^h^	17.54±1.80	<0.01^a,d,g^
						<0.001^e,h^
Nuclear markers						
p53	7.39±2.69	2.99±0.39	1.95±0.88	3.38±0.39	2.34±0.53	NS
MDM2	0.62±0.25^a^	1.10±0.29^b^	1.23±0.41	1.03±0.40^f^	2.60±0.42	<0.01^a,b^
						<0.05^f^
p21	1.59±0.40^a^	1.25±0.17^b^	1.57±0.34^e^	1.08±0.17^f^	0.54±0.14	<0.05^a,e,f^
						<0.01^b^
p27	9.37±1.33^a^	3.89±0.27^g^	3.93±0.56^c^	3.86±0.29^h^	3.46±0.29	<0.01^c,h^
						<0.001^a,g^
Cyclin D1	1.27±0.91	0.10±0.5^g^	0.21±0.13	0.040±0.01^h^	0.09±0.02	<0.05^g,h^
Ki-67	2.53±0.72^a^	0.08±0.02^g^	0.13±0.03^c^	0.06±0.03^h^	0.05±0.02	<0.001^a,g,h^
						<0.01^c^
Plasma membrane markers						
IGF1R	2.72±1.47^a^	6.34±2.62	8.48±6.17	5.18±2.47	6.67±1.62	<0.01^a^
E-cadherin	0.00±0.00	0.00±0.00	0.00±0.00	0.00±0.00	0.00±0.00	NA

ACC, adrenocortical carcinoma; ACAt, total adrenocortical adenoma; ACAc, adenoma with Cushing's syndrome; ACAn, nonfunctioning adenoma; NAG, normal adrenal gland; NA, field is not applicable.

a ACC vs NAG.

b ACAt vs NAG.

c ACAc vs ACC.

d ACAc vs ACAn.

e ACAc vs NAG.

f ACAn vs NAG.

g ACAt vs ACC.

h ACAn vs ACC.

**Table 3:** β-Catenin staining localization distribution in the different study groups.

**Localization**	**Only membrane**	**Membrane+cytoplasm**	**Membrane+cytoplasm+nucleus**
Samples			
ACC (*n*=11)	18.18% (*n*=2)	63.64% (*n*=7)	18.18% (*n*=2)
ACAt (*n*=20)	5.00% (*n*=1)	65.00% (*n*=13)	30.00% (*n*=6)
ACAc (*n*=7)	0.00% (*n*=0)	100.00% (*n*=7)	0.00% (*n*=0)
ACAn (*n*=13)	7.69% (*n*=1)	46.15% (*n*=6)	46.15% (*n*=6)
NAG (*n*=12)	100.00% (*n*=12)	0.00% (*n*=0)	0.00% (*n*=0)

ACC, adrenocortical carcinoma; ACAt, total adrenocortical adenoma; ACAc, adenoma with Cushing's syndrome; ACAn, nonfunctioning adenoma; NAG, normal adrenal gland.
